# Ten simple rules for research with humans

**DOI:** 10.1371/journal.pcbi.1013362

**Published:** 2025-08-07

**Authors:** Madeline K. Coates, Tudor V. Sava, Sarah M. Tashjian

**Affiliations:** 1 Melbourne School of Psychological Science, University of Melbourne, Parkville, Victoria, Australia; 2 Melbourne Brain Centre Imaging Unit, University of Melbourne, Parkville, Victoria, Australia; Dassault Systemes BIOVIA, UNITED STATES OF AMERICA

**What determines whether a study produces high-quality, reliable data or suffers from participant dropout, protocol violations, and compromised results?** While technical expertise and sophisticated analytical methods are essential, an often-overlooked factor significantly influences research outcomes: the quality of researcher-participant interactions. **But how many computational biologists, trained primarily in data analysis and computational methods, receive guidance in interpersonal skills that directly impact their data quality?** While standard human subjects protection training covers fundamental principles, it often lacks specific guidance for the dynamics of direct participant interaction [1].

Consider these scenarios: A genomics researcher struggles to explain complex sequencing procedures to anxious participants, leading to incomplete consent and study withdrawals. A machine learning team analyzing wearable device data discovers that participants abandoned the devices due to discomfort that could have been anticipated and addressed. A neuroimaging study loses valuable data when participants become claustrophobic during scanning because researchers failed to adequately prepare them for the experience. **What if these challenges are not inevitable costs of research, but preventable with adequate attention to the participant experience?**

Computational biology increasingly involves direct collection of human-derived data through biobanks, wearable technologies, mobile health applications, and direct participant interaction for imaging studies, genetic sampling, and behavioral assessments. Human-derived data is critical for building robust models that inform a wide range of applications including Genome-Wide Association Studies that create polygenic risk scores for conditions such as diabetes, cardiovascular disease, cancer, and autoimmune diseases [2]; microbiome profiles from human samples that aid understanding of conditions like obesity and influence national dietary recommendations [3]; randomized controlled trials that evaluate drug and treatment interventions [4]; and machine learning models analyzing real-time wearable technology data that predict health trends and directly shape consumer behavior [5]. **But how are these aims undermined when participants feel uncomfortable, misunderstood, or disengaged during data collection?**

Our framework is designed to facilitate computational biologists in effective human data collection. We offer Ten Simple Rules to help researchers build trust and rapport that translates into better data quality. We provide practical strategies to minimize participant burden while maximizing engagement. And we answer a critical question of: **What does it mean to truly empower participants as partners in scientific discovery?** We include examples informed by our experiences, participant feedback, and empirical findings from social science to provide a human-centered framework. Our goal is to stimulate the computational biology community to think about how they interact with the human sources of their data. These Ten Simple Rules provide advice for conducting studies with human participants that all researchers can benefit from, whether as a refresher for those engaging in human subjects research regularly or foundational guidance for those unfamiliar with the direct link between participant experience and data quality. These rules also apply to all research involving human subjects, whether through direct participant interaction or analysis of previously collected human data, as understanding the conditions under which data were originally gathered is essential for ensuring research validity.

## Rule 1. Build rapport

Building rapport with participants is crucial for gaining trust and creating positive research experiences. Trust and positive researcher-participant relationships directly impact data quality through improved participant disclosure rates [6], reduced attrition in longitudinal studies [7,8], and enhanced compliance and effort with protocols [9].

Computational biology presents unique rapport challenges that can severely impact data integrity. Unlike traditional clinical research where participants interact with medical professionals, computational biologists often lack training in interpersonal communication, potentially alienating participants unfamiliar with technical jargon or complex data collection procedures. Poor explanation of genomic sequencing, wearable device protocols, or longitudinal data collection can lead to selective dropout, creating systematic biases that compromise population-level modeling and reduce generalizability of computational findings.

Failure to establish rapport significantly compromises research integrity across multiple dimensions. In clinical research contexts, inadequate rapport has been linked to underreporting of sensitive health behaviors, leading to misclassification of exposure variables and attenuated effect estimates [10]. Neuroimaging studies specifically demonstrate how lack of rapport can result in participant attrition and poor data quality with no-show rates of as high as 22% and incompletion rates (among patients who showed up) as high as 3.7% [11]. Poor participant engagement leads to reduced data quality, increased missing responses, and higher dropout rates, though systematic quantification of these effects across studies remains limited. Multi-omic studies requiring multiple biological samples over time are particularly vulnerable, as attrition leads to biased estimates and can deteriorate generalizability if those who drop out differ from those who continue [12].

### Practical suggestions

#### Time.

Effective rapport-building requires approaching interactions as relaxed, friendly social exchanges where researchers demonstrate authentic interest through active listening, use appropriate language, and acknowledge the novelty of the research environment. Schedule 15–30 min of unstructured interaction time before data collection begins. While this increases session time, this investment reduces overall study costs through decreased dropout and re-collection needs.

#### Team.

Recruit team members who reflect participant demographics and can serve as cultural bridges. Consider hiring participant liaisons who share lived experiences with the study cohort. Train all staff in active listening techniques and provide scripts for explaining procedures in accessible language.

#### Environment.

Create welcoming physical spaces with comfortable seating, natural lighting, and culturally appropriate materials. For computational studies involving multiple visits, maintain consistent staff (to the extent possible) to build ongoing relationships.

#### Technology.

For studies involving wearable devices or mobile applications, provide hands-on training sessions where participants can practice with equipment in a supportive environment, reducing anxiety and improving long-term compliance. Provide video instructions participants can return to at home.

## Rule 2. Balance consent and confidentiality

Consent empowers participants by providing knowledge and choice, while confidentiality safeguards privacy by preventing unauthorized data access. Individuals are more likely to refuse to participate in biological data collection when concerned about confidentiality risks [13] and concerns about confidentiality can increase social desirability bias in self-report measures [14]. Research on participant perceptions indicates that while most research participants feel respected and listened to by study staff (85%–95%) and rate their overall experience highly (68%), only 60% feel fully prepared by the consent process [15]. The lack of adequate preparation for the research experience highlights gaps in the informed consent process despite standard ethics training.

Computational biology faces unique consent and confidentiality challenges due to the complexity of data types, long-term storage requirements, and potential for data reuse in unforeseen applications. Unlike traditional clinical studies with defined endpoints, computational research often involves biobanking, genetic sequencing, and multi-omics integration where initial consent may not cover future analytical possibilities. Participants may struggle to understand how their genomic data could be used in machine learning models, population-level analyses, or integrated with other datasets [16], making truly informed consent difficult to achieve.

Building rapport (Rule 1) can also affect perceptions of confidentiality with impacts on consent. In a study of genomic data sharing, 42.2% of participants who prioritized privacy over advancing research chose to restrict release of their data, while 56.8% of those who prioritized advancing research chose full public data release [17]. Withdrawal of consent to release data for future use limits researchers’ ability to leverage existing samples, creating problems for large-scale database efforts. Establishing rapport and clearly communicating how data are kept confidential can ameliorate participants’ concerns, potentially enhancing participants’ engagement in open data efforts.

Research with vulnerable populations requires additional care. For example, mandatory guardian involvement during consent reduces adolescent reporting of risk behaviors by up to 70%, while waiving parental consent can increase both participation rates and data accuracy [6,18]. These findings illustrate how consent requirements can inadvertently compromise data quality when they conflict with participants’ privacy concerns. Of course, sometimes individuals may not be competent to give their own consent. In those cases, guardian consent can serve as a complete proxy. In circumstances where the participant cannot give consent, self-report or behavioral data should be interpreted with appropriate caution given that lack of consent also likely reduces the validity of such data. Inadequate attention to confidentiality protections can also have devastating consequences for research programs. Poor confidentiality practices in computational studies have led to data breaches affecting thousands of participants, resulting in study termination and long-term damage to institutional research programs [19].

### Practical suggestions

#### Consent process.

Implement tiered consent procedures for vulnerable populations, obtaining guardian consent first, then participant assent when possible. Allow 24 h between consent explanation and data collection to reduce time pressure, if possible. Use visual aids and plain language summaries to explain complex data types and potential uses. Consider guardian waivers in studies with adolescents.

#### Data segmentation.

Collect sensitive information (mental health symptoms, substance use, sexual behavior) separately from others (guardians, family members, other research team members not managing consent) when ethically appropriate. Create separate consent modules for different data types (genomic, behavioral, longitudinal follow-up) allowing participants to opt into specific components.

#### Technology.

Use secure, encrypted platforms for sensitive data collection. Implement participant portals where individuals can review their consent choices and modify permissions for future data use. Provide clear data retention timelines and deletion procedures.

#### Documentation.

Maintain detailed records of who consented to what data types and uses. Create participant-friendly privacy policies explaining exactly how data will be stored, analyzed, and potentially shared. Include withdrawal procedures that specify whether data can be removed from completed analyses.

## Rule 3. Instill autonomy

Participants who experience autonomy, competence, and relatedness in the research process—core components of intrinsic motivation according to self-determination theory [20]—are likely to demonstrate higher protocol compliance and provide more complete data.

Computational biology research often involves complex, technology-mediated data collection that can feel impersonal or overwhelming to participants. Studies using ecological momentary assessment, smartphone-based data collection, continuous physiological monitoring, or digital phenotyping may require participants to integrate research activities into their daily lives for weeks or months. Unlike discrete clinical visits, these approaches demand ongoing participant engagement and decisions about when and how to participate. Participants who receive transparent communication about how their data will be used to advance scientific understanding demonstrate better long-term engagement [21].

Fostering participant autonomy becomes especially critical when individuals must carry devices and respond to prompts throughout their daily routines, as this level of integration requires active engagement rather than passive compliance. When participants feel they have meaningful control over their research experience and understand the scientific rationale behind sampling procedures, they are more likely to maintain the high levels of engagement necessary for data collection over extended periods [22].

In the case of participants who have not had exposure to research, study participation may be a valuable opportunity for self-determination. However, the novelty of the experience also renders participants vulnerable to stress and anxiety. For example, research contexts may be the first incidence of participant exposure to certain medical procedures (e.g., blood draws, stool samples, magnetic resonance imaging). If researchers navigate this situation with the required sensitivity, it can foster a positive opportunity for enhancing self-advocacy, which could yield long-term benefits for the individual and set the tone for future medical interactions. However, if the research team is not sensitive to these considerations, it could inadvertently lead to adverse outcomes. Notably, poor research experiences that enhance medical mistrust could result in participants’ reduction in seeking out future health care [23].

### Practical suggestions

#### Choice.

Offer participants choice about study participation—preferred data collection times, communication methods (text, email, phone), or optional study components. Create flexible protocols that accommodate individual schedules and preferences while maintaining scientific rigor. A participant that feels it is their choice whether to continue with a study is more likely continue and provide better data.

#### Ongoing consent.

Implement “check-in” procedures for longitudinal studies, asking participants if they want to continue and addressing any concerns. Use automated systems to remind participants of their right to withdraw without penalty and provide easy mechanisms for doing so. Including opportunities to give feedback creates a non-judgmental, empowering atmosphere that values participants’ input.

#### Transparent communication.

Explain not just what data you are collecting, but why it matters for the research questions. Provide regular updates about study progress and how participants’ contributions are being used. Use plain language summaries that avoid technical jargon.

#### Comfort monitoring.

Train staff to regularly assess participant wellbeing. Provide immediate responses to discomfort—offer breaks, snacks, water, or position changes.

## Rule 4. Facilitate STEM curiosity

Research participation provides valuable opportunities for informal STEM outreach and education that extend far beyond data collection. Participants who receive debriefings and updates about research findings show increased scientific literacy, greater trust in research, and higher likelihood of future study participation [24]. Youth participation in research design can be particularly impactful for their interest and enthusiasm in science [25]. Fostering STEM curiosity during research participation can counteract stereotypes, build scientific self-efficacy, and encourage evidence-based decision-making in participants’ daily lives.

Computational biology offers unique opportunities to spark curiosity about cutting-edge science, but researchers often miss these educational moments. Unlike traditional clinical research where procedures may seem routine, computational studies involve intersections of biology, technology, and mathematics that participants rarely encounter elsewhere. Machine learning analyses of brain imaging data, algorithmic approaches to understanding genetic variation, or real-time processing of physiological signals represent frontier science that could inspire participants—yet researchers often fail to bring participants into the fold and translate these concepts into accessible new knowledge.

Missed opportunities for STEM engagement represent significant lost potential for both individual participants and the broader scientific community. Studies with underrepresented minorities demonstrate that lack of diverse role models and poor science communication reinforce stereotypes that STEM fields are “not for people like me,” perpetuating systemic disparities in STEM participation [26]. In line with Rule 3, STEM outreach, which can occur in the research study environment, creates opportunities to increase youth motivation and attitudes toward STEM, but only if autonomy is preserved [27]. Poor science communication during research participation contributes to broader scientific illiteracy and reduced public trust in research [28]. Participants value explanation about study results, which is linked to higher willingness to participate in future research [15] and trust of research, with effects particularly high for genetic studies [29].

### Practical suggestions

#### Educational debriefing.

Allocate 10–15 min at study conclusion for accessible explanations of research goals, methods, and potential impact. Create visual aids showing how participants’ data contributes to larger scientific questions. Prepare answers to common questions about computational methods in non-technical language.

#### Diverse representation.

Intentionally recruit research staff who reflect participant demographics, particularly for studies with underrepresented populations. Highlight diverse scientists’ contributions when explaining research background and provide information about STEM career pathways relevant to participants’ interests. Exposure to female, Black, and LGBTQ+ role models in STEM can increase participants’ self-efficacy and aspirations in STEM fields [26,30,31].

#### Ongoing engagement.

Implement opt-in systems for sharing study results and broader research updates via newsletters or social media. Create participant advisory groups involving volunteers in research planning and methodology discussions where appropriate.

#### Curiosity protocols.

Develop standard talking points about study procedures that can be shared without compromising scientific integrity. For blinded studies, focus on broader research questions and computational methods rather than specific hypotheses. Encourage questions throughout the research process and train staff to respond enthusiastically to participant curiosity.

## Rule 5. Acknowledge the whole person

Research participants are more than just data points—they are individuals shaped by their unique cultural contexts and intersectional identities [32]. Participants bring to the research setting a combination of visible and invisible traits, such as race, gender, socioeconomic background, family dynamics, and personal experiences, all of which influence how they perceive and engage with the research process. Cultural norms affect comfort levels with certain procedures or perceived authority figures, while experiences with discrimination or privilege shape openness in information sharing [33].

Computational biology research often requires standardized protocols and controlled environments that may inadvertently exclude or burden participants from diverse backgrounds. Studies involving neuroimaging, genetic testing, or long-term digital monitoring may conflict with cultural practices, religious observances, or economic constraints that researchers fail to anticipate. For instance, MRI studies scheduled during Ramadan may be problematic for Muslim participants, genetic studies may raise concerns in communities with histories of research exploitation, and longitudinal app-based studies may exclude participants without reliable internet access or smartphones. Models trained on homogeneous samples produce biased algorithms that perform poorly in diverse populations, undermining the precision medicine goals that motivate much computational biology research. Research conducted primarily with WEIRD (Western, Educated, Industrialized, Rich, Democratic) populations fails to capture important biological and behavioral variation, limiting the applicability of computational models to global populations [34,35].

Participants’ willingness to engage in precision medicine research is significantly influenced by their personal and cultural experiences with biomedical research and clinical care, with historical events such as the Tuskegee Syphilis Study and the case of Henrietta Lacks contributing to mistrust of scientific research. Immigrants may face additional barriers to research participation due to unfamiliarity with navigating the medical system, language differences, and concerns about how research participation might affect their ability to receive care, highlighting the need for culturally competent communication approaches. Participants from diverse racial and ethnic backgrounds express specific concerns about the potential for precision medicine research to lead to racial profiling and discrimination, including fears about government access to patient data for non-health-related purposes and the possibility that research findings could be used to create or affirm racial stereotypes [34,35].

### Practical suggestions

#### Cultural competence.

Conduct community consultation before study design to identify potential cultural barriers or concerns. Provide multilingual materials and culturally appropriate explanations of procedures. Recruit team members who reflect participant demographics when possible.

#### Flexible scheduling.

Offer extended scheduling including evenings, weekends. Create accommodation for religious observances, work schedules, and caregiving responsibilities.

#### Accessibility planning.

Ensure physical accessibility (ramps, elevators, accessible parking) and communication accessibility (interpreters, visual aids, multiple format options). Provide comfortable spaces for caregivers or support persons. Consider transportation and offer virtual options when appropriate.

#### Holistic assessment.

Include brief screening questions about recent life stressors, sleep quality, and daily activities that might affect data collection. Document and control for these factors in analyses rather than excluding participants. Provide snacks, hydration, and comfortable environments to support participant wellbeing during longer sessions.

## Rule 6. Prepare participants for discomfort

Research environments represent novel and potentially anxiety-provoking experiences for many participants. Uncertainty amplifies feelings of loss of control and fear of the unknown, with anxiety vulnerability heightened particularly in unfamiliar settings [36]. Preparing participants for the research environment is vital because it directly addresses both psychological and physical barriers to participation. Clear, empathetic communication and comprehensive preparation help alleviate anxiety, reduce uncertainty, and build trust between participants and researchers [37,38]. When participants know what to expect, understand the reasons behind instructions, and feel supported, they are more likely to remain calm and cooperative, which not only improves their personal experience but also enhances data quality by minimizing movement and distress [37]. Moreover, a positive, well-prepared research experience can increase willingness to participate in future studies and foster greater inclusivity and diversity in research. Ultimately, thorough preparation is not only an ethical imperative that respects participants’ autonomy and well-being, but also essential for the success of research involving human subjects.

Computational biology studies often involve unfamiliar technologies and procedures that can be particularly anxiety-provoking for participants. Extended sessions in confined MRI scanners, wearing multiple physiological sensors, or interacting with novel interfaces for cognitive tasks can trigger claustrophobia, discomfort, or technology anxiety. Eye-tracking studies require participants to maintain unnatural head positions, EEG recordings involve electrode application that some find invasive, and virtual reality paradigms can cause motion sickness or disorientation [39]. Many participants have never encountered research-grade technology and may fear they will “break something expensive” or “do it wrong,” creating performance anxiety that affects natural behavior and data quality.

Inadequate preparation for discomfort leads to increased data artifacts, protocol violations, and premature study termination. Studies show that participants who feel inadequately prepared for research are more likely to withdraw early and report lower satisfaction with their experience, which can negatively impact study retention and data quality [40]. Inadequate preparation can also result in participants feeling anxious or confused about research procedures, potentially confounding study outcomes and introducing bias into the results. The scan environment has been shown to produce neural and physiological activity associated with stress, indicating that research methods themselves can introduce noise [41,42]. While adequate preparation can reduce some of these stress-related effects, researchers must still account for methodological artifacts through careful post-data processing and quality control measures. Understanding how both the research methods and the quality of participant preparation influence data outcomes is essential for generating valid and reproducible results in computational biology studies.

Regarding direct physiological effects, research shows that anxiety and stress from poor researcher interactions can alter biomarkers including cortisol levels, blood pressure, and immune function markers [41]. The ‘white coat effect’ demonstrates how participant anxiety affects cardiovascular measurements, with anxiety and outcome expectations predicting elevated blood pressure readings in medical settings [43]. In neuroimaging, stress activates neural circuits that can confound results beyond just movement artifacts, with MRI scanner environments themselves producing neural and physiological activity associated with stress [41,44]. We acknowledge this is an understudied area in computational biology specifically, which calls for future research on metrics assessing how participant interactions impact research outcomes.

### Practical suggestions

#### Preparation.

Create detailed but accessible descriptions of what participants will experience, including physical sensations, time commitments, and potential discomforts. Use photos or videos to show equipment and procedures. Schedule brief orientation visits for complex studies to familiarize participants with the environment and equipment. Allow participants to experience equipment (putting on EEG cap, lying in mock scanner) before formal testing begins.

#### Team.

Have research staff experience study procedures firsthand during training. This enables authentic descriptions of discomfort and builds empathy. Share these experiences with participants to normalize expectations and reassure them that discomfort is temporary and manageable.

#### Comfort.

Provide stress balls, breathing exercises, or other comfort aids as appropriate. For long sessions, offer scheduled breaks, position changes, and refreshments. Train staff to recognize signs of distress and respond supportively without compromising data collection.

## Rule 7. Appropriately train the team

Comprehensive team training is essential for maintaining participant safety, ensuring data quality, and upholding ethical standards in human subjects research. All team members must understand study protocols and demonstrate competence in working with specific populations, including awareness of cultural, psychological, and physical requirements [45]. Training requirements vary by population—studies with minors often require background checks and specialized certifications, while genetic research may mandate genetic counseling expertise.

Computational biology teams face unique training challenges because they often lack formal preparation for human interaction despite increasingly direct participant contact. Unlike clinical research teams with medical training backgrounds, computational researchers may have technical expertise but minimal experience with participant psychology, cultural sensitivity, or crisis management. Studies involving brain-computer interfaces require staff trained in both neurotechnology and participant safety protocols, while research using ambulatory monitoring devices demands expertise in troubleshooting technical issues while maintaining participant comfort. Many computational biology studies involve prolonged data collection sessions or complex multi-modal recordings that require specialized skills in managing participant fatigue, equipment troubleshooting, and emergency procedures.

Inadequately trained teams create significant risks for both participants and research integrity. Poor staff training and unsupportive supervision have been associated with increased emotional distress among both staff and participants, as untrained staff may misinterpret ethical guidelines or fail to respond appropriately to participant needs [46]. The literature emphasizes that researchers and research teams must value diversity, acquire cultural knowledge, and adapt to the cultural contexts of their participants to ensure effective interactions and appropriate engagement in study design and implementation [47]. Studies have found that individuals who received cultural competence training scored significantly higher on measures of cultural knowledge, skills, and awareness, which are linked to higher quality research outcomes and improved participant trust and retention [48]. Research on informed consent processes highlights that inadequate education and training of research staff are major contributors to deficiencies in conveying risks, benefits, and procedures to participants, which can compromise informed consent and increase legal risk [49]. Inadequate staff training is also identified as a factor that can undermine reproducibility, rigor, and transparency in research, particularly when protocols are not clearly explained or followed [50].

### Practical suggestions

#### Specialized training.

Require population-specific training for all team members—working with children certifications for youth studies, cultural competency training for diverse populations, or genetic counseling certification for genomic research. Ensure at least one team member has crisis intervention training for studies involving mental health assessments or potentially distressing medical procedures.

#### Technical competency.

Train staff in both technical procedures and participant communication about those procedures. Require hands-on practice with all equipment and protocols before working with participants. Develop protocols that maintain participant comfort during technical difficulties.

#### Ongoing development.

Implement regular team meetings to discuss challenging situations and share best practices. Provide continuing education opportunities and peer consultation for complex cases. Monitor team burnout and provide support for staff working with sensitive populations or traumatic content.

#### Documentation and supervision.

Maintain detailed training records and competency assessments for all staff. Establish clear supervision hierarchies with experienced researchers overseeing newer team members. Create standardized response protocols for common situations like participant distress, equipment malfunction, or disclosure of sensitive information.

## Rule 8. Create a research partnership

Research partnerships involve actively engaging participants in shaping the research process, recognizing their agency as key stakeholders rather than passive subjects. This collaborative approach allows participants to contribute ideas and feedback, ensuring their perspectives are meaningfully considered throughout the research lifecycle [25]. Evidence shows that community engagement in research increases the relevance and uptake of findings, as demonstrated by case examples where research agendas set in collaboration with communities addressed self-identified needs and led to sustainable changes [51].

Computational biology research often involves complex algorithms, data integration, and modeling approaches that can seem abstract or disconnected from participants’ lived experiences. Studies developing personalized algorithms, population health models, or digital health interventions may fail to address participants’ actual priorities or concerns without meaningful input. Researchers focused on technical innovation may design elegant computational solutions that prove impractical in real-world implementation because they lack participant insight into daily life constraints, cultural preferences, or usability concerns.

Failure to establish genuine research partnerships leads to reduced participant engagement, compromised data quality, and limited real-world impact. Research conducted without community input often fails to address questions that are most relevant to the populations affected, resulting in findings with limited practical applicability and minimal influence on policy or practice. Digital health interventions that do not adequately consider participant needs and usability in their design are associated with lower engagement and higher dropout rates, highlighting the importance of user-centered platform features to support participant retention in clinical research [52]. Computational models trained on data from disengaged participants may overlook important behavioral patterns and contextual factors, ultimately reducing model performance and generalizability in real-world deployment.

### Practical suggestions

#### Co-design.

Establish participant advisory groups early in study planning to review research questions, methods, and procedures. Compensate participants fairly for their time and expertise in these advisory roles.

#### Feedback.

Create structured mechanisms for ongoing participant input throughout the study, not just at the beginning. Implement regular check-ins asking about study burden, procedure clarity, and suggested improvements.

#### Collaborative analysis.

When appropriate, involve participant representatives in data interpretation discussions. Their insights can reveal patterns or explanations that researchers might miss.

## Rule 9. Don’t neglect (non-participant) stakeholders

Successful human research extends beyond the participant-researcher relationship to include a broader ecosystem of stakeholders whose support, expertise, and trust are essential for research integrity and impact. Key stakeholders include ethics boards, community representatives, healthcare providers, technology vendors, regulatory agencies, and policymakers who influence research feasibility, credibility, and translation. Effective stakeholder engagement ensures research addresses real-world needs, maintains public trust, has effective implementation, and is not unduly held up in regulatory hurdles.

Computational biology research involves particularly complex stakeholder networks due to its interdisciplinary nature and reliance on technologies and infrastructure. Studies may require collaboration with hospital IT departments for electronic health record access, partnerships with technology companies for device development, and engagement with regulatory bodies for algorithm validation. Research involving cloud computing platforms, artificial intelligence systems, or federated learning approaches requires coordination with data security experts and legal teams to ensure compliance with evolving privacy regulations. Unlike traditional clinical research with established stakeholder relationships, computational biology often requires building new partnerships across sectors that may have different priorities, timelines, and success metrics.

When stakeholders are excluded from research design, studies risk prioritizing investigator-driven questions over issues that address real-world health disparities or practical care gaps, reducing translational impact. Disengaged stakeholders may perceive research as extractive, leading to recruitment challenges, higher dropout rates, and biased samples that undermine generalizability [53]. When stakeholders are not meaningfully involved, there is often diminished trust, less willingness to share data, and reduced support for research activities, all of which can impair recruitment, limit access to critical information, and ultimately threaten the feasibility and generalizability of studies [54]. Without stakeholder collaboration to address data silos and privacy concerns, institutions cannot provide the sufficient data access needed for machine learning to reach its full potential [55]. Furthermore, computational models developed without input from domain experts and end-users often fail to align with clinical decision-making processes, resulting in sophisticated algorithms that remain unused despite their technical merit [56].

### Practical suggestions

#### Stakeholder mapping.

Identify all relevant stakeholders early in study planning, including regulatory bodies, technology partners, healthcare systems, community organizations, and potential end-users of research findings. Create stakeholder engagement plans with specific communication strategies and timelines for each group.

#### Regular communication.

Establish quarterly stakeholder meetings or updates tailored to each group’s interests and expertise level. Provide technical stakeholders with detailed methodology information while offering policymakers concise summaries focused on implications and applications.

#### Collaborative planning.

Involve key stakeholders in study design discussions to identify potential barriers and ensure feasibility. Engage technology partners in protocol development to ensure data collection systems can meet research requirements while maintaining security standards. Include healthcare providers in recruitment strategies to leverage their patient relationships and clinical insight. Develop contingency plans for common stakeholder issues such as technology failures, regulatory changes, or community concerns about data privacy.

## Rule 10. Individualize the experience

Personalizing the research experience represents the culmination of effective human subjects research, requiring integration of all previous rules to tailor interactions to individual participant needs, preferences, and circumstances. Successful individualization requires willingness to adapt procedures while maintaining scientific rigor. Individualized approaches result in higher participation and retention rates [57] as do providing autonomy (Rule 3), choice (Rules 2/3), and appropriate incentives (Rule 10) [7,8].

Computational biology research often involves standardized protocols and automated data collection systems that can feel impersonal and inflexible to participants. When participants experience poor person-protocol fit, they provide lower quality data. Studies using intensive longitudinal designs, ecological momentary assessment, or multi-modal data collection may require participants to engage with technology for weeks or months, making personalization crucial for sustained participation. Digital phenotyping studies that collect smartphone data, wearable sensor measurements, or repeated online assessments must balance standardization for analytical validity with customization for user experience. Participants vary widely in their technology comfort, cognitive load tolerance, and motivation for sustained engagement, requiring adaptive approaches that traditional research protocols may not accommodate.

Personal sensing technologies, such as smartphones and wearables, enable continuous and passive collection of behavioral and physiological data, offering new opportunities to tailor research protocols to individual needs and contexts. However, personalization is challenged by the high variability in how individuals use technology, differences in device sensors, and the need to balance unobtrusive data collection with participant privacy and autonomy. To maximize both scientific validity and participant engagement, adaptive research designs must incorporate user-centric approaches that provide participants with meaningful control over their data and ensure transparency about how personal information is used.

### Practical suggestions

#### Pre-session assessment.

Conduct brief surveys to understand participant preferences, technology experience, comfort levels, and potential concerns. Ask about previous research experience, preferred communication styles, and any accommodations needed. Use this information to customize the session approach while maintaining protocol integrity.

#### Adaptive protocols.

Build flexibility into study procedures allowing for individualized pacing, break schedules, and task ordering. Train staff to recognize signs of fatigue, anxiety, or disengagement and respond appropriately. Develop decision trees for common scenarios (e.g., technology difficulties, participant distress, attention issues) that maintain data quality while supporting participant needs.

#### Individualized motivation.

Offer diverse compensation options aligned with participant values—monetary payments, personalized research outputs (brain images, genetic summaries), learning opportunities, or contribution certificates. Provide real-time feedback about data quality and study progress as appropriate.

## Conclusions

**Fig 1:**
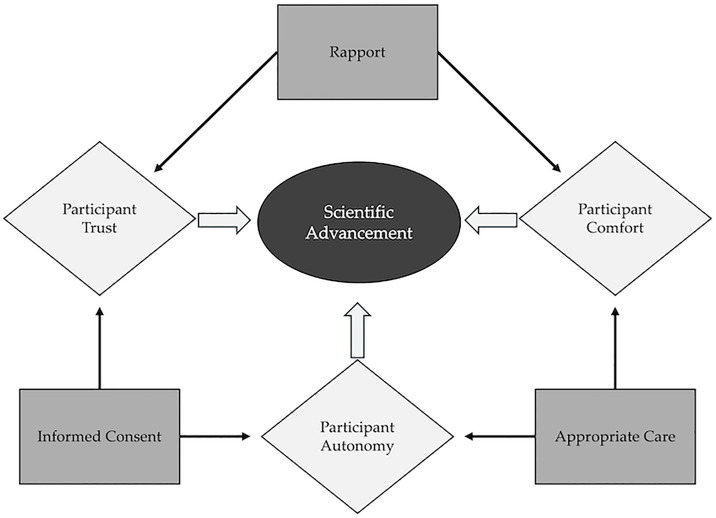
Schematic of the interplay between participant experience (diamonds) and researcher considerations (rectangles) representing the dynamic bidirectional interplay that ultimately leads to scientific advancement (oval). Participant experience directly impacts scientific advancement and is influenced by interactions with the research team.

These rules are not independent recommendations but form an integrated framework. Rule 1 (rapport) enables Rule 10 (individualization), while Rule 7 (team training) supports effective implementation of Rules 2–6. Like the Rules themselves, researchers and participants are not separate in the scientific process and the dynamics between these entities should be considered as a system (Fig 1). Our hope is that these Rules will prompt efforts to engage with human participants effectively and facilitate more constructive human research.

These Ten Simple Rules draw from converging evidence across multiple disciplines. Psychology research demonstrates how rapport and autonomy enhance participant engagement and data quality (Rules 1, 3), while ethical frameworks emphasize respect for persons and beneficence (Rules 2, 5). Legal requirements for informed consent align with psychological findings on preparation and understanding (Rules 2, 6). Experimental design principles support our emphasis on training and standardization (Rule 7), while philosophical approaches to participatory research inform our partnership models (Rules 8, 9). Rather than contradicting one another, these disciplines provide complementary perspectives that collectively support human-centered research practices. We recognize that implementing these rules requires institutional investment in training, time, and personnel that may seem at odds with productivity pressures. Additionally, computational biology’s interdisciplinary nature creates unique challenges, including scalability concerns. These Rules are not prescriptive or exhaustive, rather they serve as a call for researchers to examine how their actions, interactions, and study design impact the quality of human data.

Finally, the goal of these Ten Simple Rules is not to reinvent ethical principles but to translate established knowledge into actionable guidance for a community that needs specific support in conducting high-quality human subjects research. The computational biology community represents an intersection of technical expertise and human subjects research that requires specialized guidance. Traditional biomedical research training may not adequately prepare computational researchers for the specific challenges they face in human subjects research, such as explaining complex analytical methods to participants, managing expectations about data use, or ensuring meaningful consent for novel computational approaches. Interdisciplinary collaboration between computational biologists, social scientists, and ethicists could yield more sophisticated approaches to human subjects research in computational contexts.

Notably little research has been done on the effects of researcher-participant interactions and the integrity of computational biology studies. While individual components such as rapport, consent processes, and participant preparation have been studied in clinical and psychological research settings, their specific application to computational biology contexts and their combined effects on data quality in technology-mediated research environments remain largely unexamined. Much of the research cited herein pertains to qualitative studies, which have less relevance for computational biologists. Future research should develop metrics for assessing the impact of improved participant interactions on research outcomes and assess training curricula for computational biology teams transitioning to human subjects work. Research is needed on participant advisory board models specifically adapted for computational research contexts as well as longitudinal work on whether improved participant experience translates to better long-term research outcomes. We encourage computational biology programs to integrate human subjects interaction training into graduate curricula and funding agencies to consider participant experience metrics when evaluating research proposals.

## References

[1] Metcalf J, Crawford K. Where are human subjects in Big Data research? The emerging ethics divide. Big Data Society. 2016;3(1). doi: 10.1177/2053951716650211

[2] Uffelmann E, Huang QQ, Munung NS, de Vries J, Okada Y, Martin AR. Genome-wide association studies. Nat Rev Methods Primers. 2021;1.

[3] Gilbert JA, Blaser MJ, Caporaso JG, Jansson JK, Lynch SV, Knight R. Current understanding of the human microbiome. Nat Med. 2018;24(4):392–400. doi: 10.1038/nm.4517 29634682 PMC7043356

[4] Hariton E, Locascio JJ. Randomised controlled trials—the gold standard for effectiveness research: Study design: randomised controlled trials. BJOG. 2018;125(13):1716. doi: 10.1111/1471-0528.15199 29916205 PMC6235704

[5] Varoquaux G, Colliot O. Evaluating machine learning models and their diagnostic value. Neuromethods. 2023. 37988512

[6] Rojas NL, Sherrit L, Harris S, Knight JR. The role of parental consent in adolescent substance use research. J Adolesc Health. 2008;42(2):192–7. doi: 10.1016/j.jadohealth.2007.07.011 18207098

[7] Jong ST, Stevenson R, Winpenny EM, Corder K, van Sluijs EMF. Recruitment and retention into longitudinal health research from an adolescent perspective: a qualitative study. BMC Med Res Methodol. 2023;23(1):16. doi: 10.1186/s12874-022-01802-7 36647003 PMC9841671

[8] Gabel M, Bollinger RM, Coble DW, Grill JD, Edwards DF, Lingler JH, et al. Retaining participants in longitudinal studies of Alzheimer’s disease. J Alzheimers Dis. 2022;87(2):945–55. doi: 10.3233/JAD-215710 35404282 PMC9673904

[9] Curry SM, Gravina NE, Sleiman AA, Richard E. The effects of engaging in rapport-building behaviors on productivity and discretionary effort. J Organ Behav Manage. 2019;39(3–4).

[10] Durant LE, Carey MP, Schroder KEE. Effects of anonymity, gender, and erotophilia on the quality of data obtained from self-reports of socially sensitive behaviors. J Behav Med. 2002;25(5). 10.1023/a:1020419023766 PMC2430657 12442560

[11] Norbash A, Yucel K, Yuh W, Doros G, Ajam A, Lang E, et al. Effect of team training on improving MRI study completion rates and no-show rates. J Magn Reson Imaging. 2016;44(4):1040–7. doi: 10.1002/jmri.25219 27126735 PMC5612491

[12] Gustavson K, von Soest T, Karevold E, Røysamb E. Attrition and generalizability in longitudinal studies: findings from a 15-year population-based study and a Monte Carlo simulation study. BMC Public Health. 2012;12:918. doi: 10.1186/1471-2458-12-918 23107281 PMC3503744

[13] Siminoff LA, Wilson-Genderson M, Mosavel M, Barker L, Trgina J, Traino HM. Confidentiality in biobanking research: a comparison of donor and nondonor families’ understanding of risks. Genet Test Mol Biomarkers. 2017;21(3):171–7. doi: 10.1089/gtmb.2016.0407 28121471 PMC5367914

[14] Perinelli E, Gremigni P. Use of social desirability scales in clinical psychology: a systematic review. J Clin Psychol. 2016;72(6):534–51. doi: 10.1002/jclp.22284 26970350

[15] Kost RG, Andrews J, Chatterjee R, Cheng AC, Connally L, Dozier A. What research participants say about their research experiences in empowering the participant voice: outcomes and actionable data. J Clin Transl Sci. 2025;9(1):e43. 10.1017/cts.2025.3 PMC11930633 40129933

[16] Shabani M, Knoppers BM, Borry P. From the principles of genomic data sharing to the practices of data access committees. EMBO Mol Med. 2015;7(5). 10.15252/emmm.201405002 PMC4492813 25759363

[17] Oliver JM, Slashinski MJ, Wang T, Kelly PA, Hilsenbeck SG, McGuire AL. Balancing the risks and benefits of genomic data sharing: genome research participants’ perspectives. Public Health Genomics. 2012;15. 10.1159/000334718 PMC3318928 22213783

[18] Wasilewski S. Ethical considerations for requesting waivers of parental consent for research with minor adolescents who identify as LGBTQ. Ethics Behav. 2024;34(3).

[19] Gymrek M, McGuire AL, Golan D, Halperin E, Erlich Y. Identifying personal genomes by surname inference. Science. 2013;339(6117). 10.1126/science.1229566 23329047

[20] Ryan RM, Deci EL. Self-determination theory and the facilitation of intrinsic motivation, social development, and well-being. Am Psychol. 2000;55(1):68–78. doi: 10.1037//0003-066x.55.1.68 11392867

[21] Huckvale K, Venkatesh S, Christensen H. Toward clinical digital phenotyping: a timely opportunity to consider purpose, quality, and safety. npj Digital Medicine. 2019;2. 10.1038/s41746-019-0166-1 PMC6731256 31508498

[22] Stone AA, Shiffman S. Capturing momentary, self-report data: a proposal for reporting guidelines. Ann Behav Med. 2002;24(3):236–43. doi: 10.1207/S15324796ABM2403_09 12173681

[23] LaVeist TA, Isaac LA, Williams KP. Mistrust of health care organizations is associated with underutilization of health services. Health Serv Res. 2009;44(6):2093–105. doi: 10.1111/j.1475-6773.2009.01017.x 19732170 PMC2796316

[24] Habig B, Gupta P. Authentic STEM research, practices of science, and interest development in an informal science education program. Int J STEM Educ. 2021;8(1).

[25] Hawke LD, Relihan J, Miller J, McCann E, Rong J, Darnay K. Engaging youth in research planning, design and execution: practical recommendations for researchers. Health Expectations. 2018;21. 10.1111/hex.12795 PMC6250868 29858526

[26] Cheryan S, Master A, Meltzoff AN. Cultural stereotypes as gatekeepers: increasing girls’ interest in computer science and engineering by diversifying stereotypes. Front Psychol. 2015;6:49. doi: 10.3389/fpsyg.2015.00049 25717308 PMC4323745

[27] Vennix J, den Brok P, Taconis R. Do outreach activities in secondary STEM education motivate students and improve their attitudes towards STEM?. Int J Sci Educ. 2018;40(11).

[28] Freeman JB. Measuring and resolving LGBTQ disparities in STEM. Policy Insights Behav Brain Sci. 2020;7(2):141–8. doi: 10.1177/2372732220943232

[29] Wilkins CH, Mapes BM, Jerome RN, Villalta-Gil V, Pulley JM, Harris PA. Understanding what information is valued by research participants, and why. Health Aff. 2019;38(3). 10.1377/hlthaff.2018.05046 PMC6706772 30830824

[30] Hampton-Marcell J, Bryson T, Larson J, Childers T, Pasero S, Watkins C. Leveraging national laboratories to increase Black representation in STEM: recommendations within the Department of Energy. Int J STEM Educ. 2023;10(1).

[31] Lockwood P. “Someone like me can be successful”: Do college students need same-gender role models?. Psychol Women Q. 2006;30(1).

[32] Guidelines on multicultural education, training, research, practice, and organizational change for psychologists. Am Psychologist. 2003;58. 10.1037/0003-066x.58.5.377 12971086

[33] George S, Duran N, Norris K. A systematic review of barriers and facilitators to minority research participation among African Americans, Latinos, Asian Americans, and Pacific Islanders. Am J Public Health. 2014;104. 10.2105/AJPH.2013.301706 PMC3935672 24328648

[34] Chiao JY, Cheon BK. The weirdest brains in the world. Behav Brain Sci. 2010;33. 10.1017/S0140525X10000282 20546651

[35] Henrich J, Heine SJ, Norenzayan A. Most people are not WEIRD. Nature. 2010;466. 10.1038/466029a 20595995

[36] Grupe DW, Nitschke JB. Uncertainty and anticipation in anxiety: an integrated neurobiological and psychological perspective. Nat Rev Neurosci. 2013;14. 10.1038/nrn3524 PMC4276319 23783199

[37] Dewey RS, Ward C, Junor A, Horobin A. Talk to us! Communication is a key factor in improving the comfort of MRI research participants. Health Expect. 2021;24(4):1137–44. doi: 10.1111/hex.13217 33949066 PMC8369077

[38] Kraft SA, Cho MK, Gillespie K, Halley M, Varsava N, Ormond KE, et al. Beyond consent: building trusting relationships with diverse populations in precision medicine research. Am J Bioethics. 2018;18(4). 10.1080/15265161.2018.1431322 PMC6173191 29621457

[39] Saredakis D, Szpak A, Birckhead B, Keage HAD, Rizzo A, Loetscher T. Factors associated with virtual reality sickness in head-mounted displays: a systematic review and meta-analysis. Front Hum Neurosci. 2020;14:96. doi: 10.3389/fnhum.2020.00096 32300295 PMC7145389

[40] Terceiro L, Mustafa MI, Hägglund M, Kharko A. Research participants’ engagement and retention in digital health interventions research: protocol for mixed methods systematic review. JMIR Res Protoc. 2025;14:e65099. 10.2196/65099 PMC11748419 39752662

[41] Muehlhan M, Lueken U, Wittchen H-U, Kirschbaum C. The scanner as a stressor: evidence from subjective and neuroendocrine stress parameters in the time course of a functional magnetic resonance imaging session. Int J Psychophysiol. 2011;79(2):118–26. doi: 10.1016/j.ijpsycho.2010.09.009 20875462

[42] Tessner KD, Walker EF, Hochman K, Hamann S. Cortisol responses of healthy volunteers undergoing magnetic resonance imaging. Hum Brain Mapp. 2006;27(11):889–95. doi: 10.1002/hbm.20229 16544325 PMC6871496

[43] Jhalani J, Goyal T, Clemow L, Schwartz JE, Pickering TG, Gerin W. Anxiety and outcome expectations predict the white-coat effect. Blood Pressure Monit. 2005. 10.1097/00126097-200512000-00006 16496447

[44] Gossett EW, Wheelock MD, Goodman AM, Orem TR, Harnett NG, Wood KH, et al. Anticipatory stress associated with functional magnetic resonance imaging: Implications for psychosocial stress research. Int J Psychophysiol. 2018;125:35–41. doi: 10.1016/j.ijpsycho.2018.02.005 29454000 PMC5842805

[45] Antle AN. The ethics of doing research with vulnerable populations. Interactions. 2017;24(6):74–7. doi: 10.1145/3137107

[46] Nguyen M, Goldsamt L, Mazibuko N, Zondo S, Fielding-Miller R. Emotional distress among frontline research staff. Soc Sci Med. 2021;281. 10.1016/j.socscimed.2021.114101 PMC10484114 34120087

[47] Villagran MAL. Cultural competence in research. School Information Student Res J. 2022;12(1).

[48] Dunaway KE, Morrow JA, Porter BE. Development and validation of the cultural competence of program evaluators (CCPE) self-report scale. Am J Eval. 2012;33(4):496–514. doi: 10.1177/1098214012445280

[49] Nusbaum L, Douglas B, Damus K, Paasche-Orlow M, Estrella-Luna N. Communicating risks and benefits in informed consent for research: a qualitative study. Glob Qual Nurs Res. 2017;4:2333393617732017. doi: 10.1177/2333393617732017 28975139 PMC5613795

[50] Six factors affecting reproducibility in life science research and how to handle them. Nature Portfolio. 2019.

[51] Holzer JK, Ellis L, Merritt MW. Why we need community engagement in medical research. J Investig Med. 2014;62(6):851–5. doi: 10.1097/JIM.0000000000000097 24979468 PMC4108547

[52] Terceiro L, Mustafa MI, Hägglund M, Kharko A. Research participants’ engagement and retention in digital health interventions research: protocol for mixed methods systematic review. JMIR Res Protocols. 2025;14:e65099. 10.2196/65099 PMC11748419 39752662

[53] Concannon TW, Meissner P, Grunbaum JA, McElwee N, Guise JM, Santa J. A new taxonomy for stakeholder engagement in patient-centered outcomes research. J General Intern Med. 2012;27. 10.1007/s11606-012-2037-1 PMC3403141 22528615

[54] Shelton RC, Cooper BR, Stirman SW. The sustainability of evidence-based interventions and practices in public health and health care. Annu Rev Public Health. 2018. 10.1146/annurev-publhealth-040617-014731 29328872

[55] Rieke N, Hancox J, Li W, Milletarì F, Roth HR, Albarqouni S, et al. The future of digital health with federated learning. NPJ Digit Med. 2020;3:119. doi: 10.1038/s41746-020-00323-1 33015372 PMC7490367

[56] Laurisz N, Ćwiklicki M, Żabiński M, Canestrino R, Magliocca P. The stakeholders’ involvement in healthcare 4.0 services provision: The perspective of co-creation. Int J Environ Res Public Health. 2023;20(3). 10.3390/ijerph20032416 PMC9914953 36767782

[57] Abshire M, Dinglas VD, Cajita MIA, Eakin MN, Needham DM, Himmelfarb CD. Participant retention practices in longitudinal clinical research studies with high retention rates. BMC Med Res Methodol. 2017;17(1):30. doi: 10.1186/s12874-017-0310-z 28219336 PMC5319074

